# An Atypical Case of Infectious Myositis in a Young Woman on Immunosuppressive Therapy

**DOI:** 10.7759/cureus.86545

**Published:** 2025-06-22

**Authors:** Jamie Therese Abad, Sreenavya Gandikota, Allen Chehimi, Connor Bunch, Diana Jomaa

**Affiliations:** 1 Internal Medicine, Henry Ford Health System, Detroit, USA; 2 Internal Medicine, Wayne State University School of Medicine, Detroit, USA; 3 Internal Medicine, Michigan State University College of Human Medicine, East Lansing, USA; 4 Emergency Medicine and Internal Medicine, Henry Ford Health System, Detroit, USA

**Keywords:** acute myositis, bacterial myositis, bacterial pyomyositis, infectious myositis, pyomyositis, soft tissue infection

## Abstract

Infectious myositis is a rare but serious condition typically caused by bacterial pathogens. In immunocompromised patients, including those on long-term immunosuppressive therapy, clinical signs of myositis can be subtle or delayed. We present the case of a 21-year-old woman with systemic lupus erythematosus (SLE) on immunosuppressive therapy who presented with pain, fever, tachycardia, and swelling of the right lower leg. Initial evaluation revealed no skin defects or rash and normal creatine phosphokinase (CPK) levels. A non-contrast computed tomography (CT) scan of her leg showed some soft tissue changes, but it was only after a week of worsening symptoms that contrast-enhanced CT imaging revealed a multiloculated, large abscess, measuring 23.7 cm in length, in the anterior compartment of the leg. The abscess was drained surgically, and intraoperative cultures grew methicillin-resistant *Staphylococcus aureus*. The absence of early definitive findings, including a normal CPK level, may have contributed to the delay in diagnosis.

This case highlights the diagnostic challenges of infectious myositis in immunosuppressed patients, where early imaging and laboratory findings can be misleading, underscoring the importance of repeated clinical assessment and timely advanced imaging to ensure early detection and appropriate treatment.

## Introduction

Infectious myositis is a rare infection of the skeletal muscles caused by a variety of pathogens, including bacteria, fungi, parasites, and viruses. Unlike more superficial soft tissue infections, such as cellulitis (which affects the skin and subcutaneous tissue) or necrotizing fasciitis (which involves the fascia), infectious myositis affects the muscle itself. *Staphylococcus aureus* is the most common bacterial cause of myositis, particularly methicillin-resistant strains, although group A streptococci, *Clostridium* species, and other organisms have also been implicated [1,2]. Bacterial myositis can develop through direct inoculation, contiguous infectious spread from nearby structures, or hematogenous dissemination from a distant source. Classic symptoms include localized muscle pain, swelling, and systemic symptoms such as fever and malaise.

Given the potential for severe complications, infectious myositis is best understood as a broad clinical spectrum, which can include pyomyositis, a localized bacterial infection with abscess formation, and necrotizing myositis, a rare but rapidly progressive condition characterized by diffuse muscle necrosis. Timely diagnosis and prompt initiation of appropriate medical therapy are critical, especially in immunocompromised individuals who are at a higher risk for more severe disease [3]. In such patients, typical signs of infection, such as fever and leukocytosis, may be diminished or absent due to a blunted immune response, which can delay recognition.

Here, we present a case of infectious myositis in a young adult on immunosuppressive therapy who had a delayed diagnosis due to the lack of typical clinical and imaging findings normally associated with deep muscle infection. This case illustrates the challenges in identifying infectious myositis in immunocompromised patients, including the limitations of standard diagnostic markers such as creatine phosphokinase (CPK) and white blood cell (WBC) counts, which may appear normal despite active infection. These diagnostic limitations underscore the critical need for clinical vigilance and timely reassessment to avoid severe complications.

## Case presentation

A 21-year-old woman with systemic lupus erythematosus (SLE) presented with fever along with pain and swelling in her right lower leg for two days. She reported having had a mechanical fall one week prior, followed by progressive difficulty bearing weight on the affected leg. She denied any other trauma, skin wounds, penetrating injuries, or recent infections prior to symptom onset.

She was diagnosed with SLE several years prior, initially presenting with fatigue, a malar rash, and inflammatory arthritis. She had been maintained on prednisone since diagnosis and, at the time of presentation, was taking 10 mg daily along with hydroxychloroquine 200 mg daily. Regarding prior therapies, she was treated with mycophenolate mofetil approximately three years earlier for presumed lupus nephritis, which improved with immunosuppression, although no kidney biopsy was obtained. Mycophenolate was later discontinued due to tolerability issues, and she was subsequently transitioned to azathioprine, which was discontinued more recently due to leukopenia.

She had no history of recurrent infections, intravenous (IV) drug use, or recent travel. She also had no history of diabetes mellitus, human immunodeficiency virus (HIV) infection, malignancy, or other known causes of immunodeficiency beyond her SLE and immunosuppressive therapy. On presentation, she had an elevated temperature of 39.3°C and tachycardia with a heart rate of 140 beats per minute. Physical examination revealed swelling of her right lower extremity and tenderness to palpation with no visible overlying skin abnormalities or defects. On neuromuscular examination, she had a full range of motion in all limbs with no obvious sensory or motor deficits.

Initial laboratory evaluation revealed a normal white blood cell count, elevated creatinine, normal creatine phosphokinase, and elevated inflammatory markers, including C-reactive protein (CRP), erythrocyte sedimentation rate (ESR), and D-dimer (Table 1). Urinalysis was notable for positive leukocyte esterase, moderate blood, and the presence of bacteria, consistent with a urinary tract infection (UTI). Blood cultures were negative for bacteremia.

**Table 1:** Initial laboratory findings on presentation Bold values indicate elevated inflammatory markers. WBC: white blood cell, ESR: erythrocyte sedimentation rate, CRP: C-reactive protein, FEU: fibrinogen equivalent unit, CPK: creatine phosphokinase

Test	Result	Reference range
WBC count	4.1 K/uL	3.8-10.6 K/uL
Hemoglobin	8.8 g/dL	12.0-15.0 g/dL
Hematocrit	27.3%	36%-46%
Mean corpuscular volume	75.1 fL	80-100 fL
Creatinine	3.17 mg/dL	<1.03 mg/dL
ESR	72 mm/hour	<20 mm/hour
CRP	12.3 mg/dL	<0.5 mg/dL
D-dimer	4.19 ug/mL FEU	<0.50 ug/mL FEU
CPK	125 IU/L	<178 IU/L

**Figure 1:**
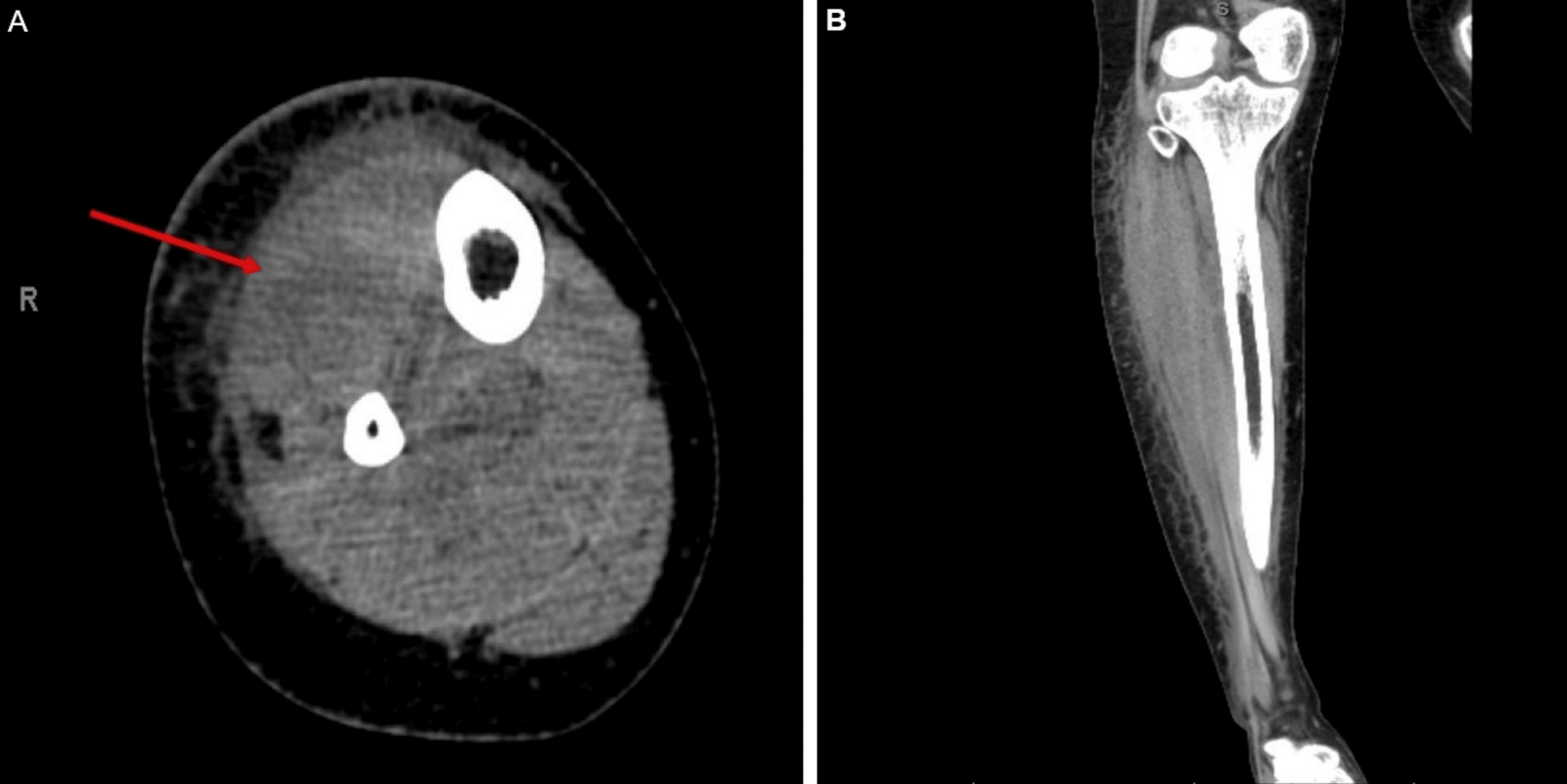
Non-contrast CT of the right lower extremity showing nonspecific soft tissue changes. Axial (A) and coronal (B) non-contrast CT images of the right lower extremity demonstrate subcutaneous edema and heterogenous hypodensity within the anterior muscle compartment, concerning for muscle atrophy or early inflammation. The red arrow in A highlights the anterior compartment with blurring of fascial planes, suggesting early infectious myositis. No discrete fluid collection is seen. Osseous structures are intact without signs of periostitis, endosteal scalloping, or radiopaque foreign bodies. CT: computed tomography

Radiography of the tibia and fibula showed no evidence of acute trauma or abnormalities. Non-contrast computed tomography (CT) of the right lower extremity revealed subcutaneous edema, heterogenous hypodensity consistent with muscle atrophy, and blurring of the fascial planes within the anterior compartment. These findings were concerning for early myositis or evolving compartment syndrome (Figure 1). Doppler ultrasonography did not show any acute thromboembolic processes. Surgical evaluation ruled out compartment syndrome and indicated no need for surgical intervention.

She was started on intravenous ceftriaxone for the UTI and admitted for further evaluation. Urine cultures later grew >100,000 colony-forming units (CFU) per mL *Staphylococcus aureus *and >100,000 CFU per mL *Streptococcus agalactiae* (group B strep). Nephrology specialists were consulted due to concern for lupus nephritis in light of the new acute kidney injury. A biopsy of the kidney was initially planned; however, the kidney injury resolved with intravenous fluids, suggesting a pre-renal injury etiology. Other laboratory testing revealed positive anti-double-stranded DNA (anti-dsDNA) antibodies (>1:320 titer), positive ribonucleoprotein antibodies (>8.0 enzyme-linked immunosorbent assay (ELISA) units), positive Smith antibodies (>8.0 ELISA units), low C3 complement (39 mg/dL), and low C4 complement (<8 mg/dL), which raised concern for an active lupus flare. Her steroid dose was increased with plans to taper toward discharge.

**Figure 2:**
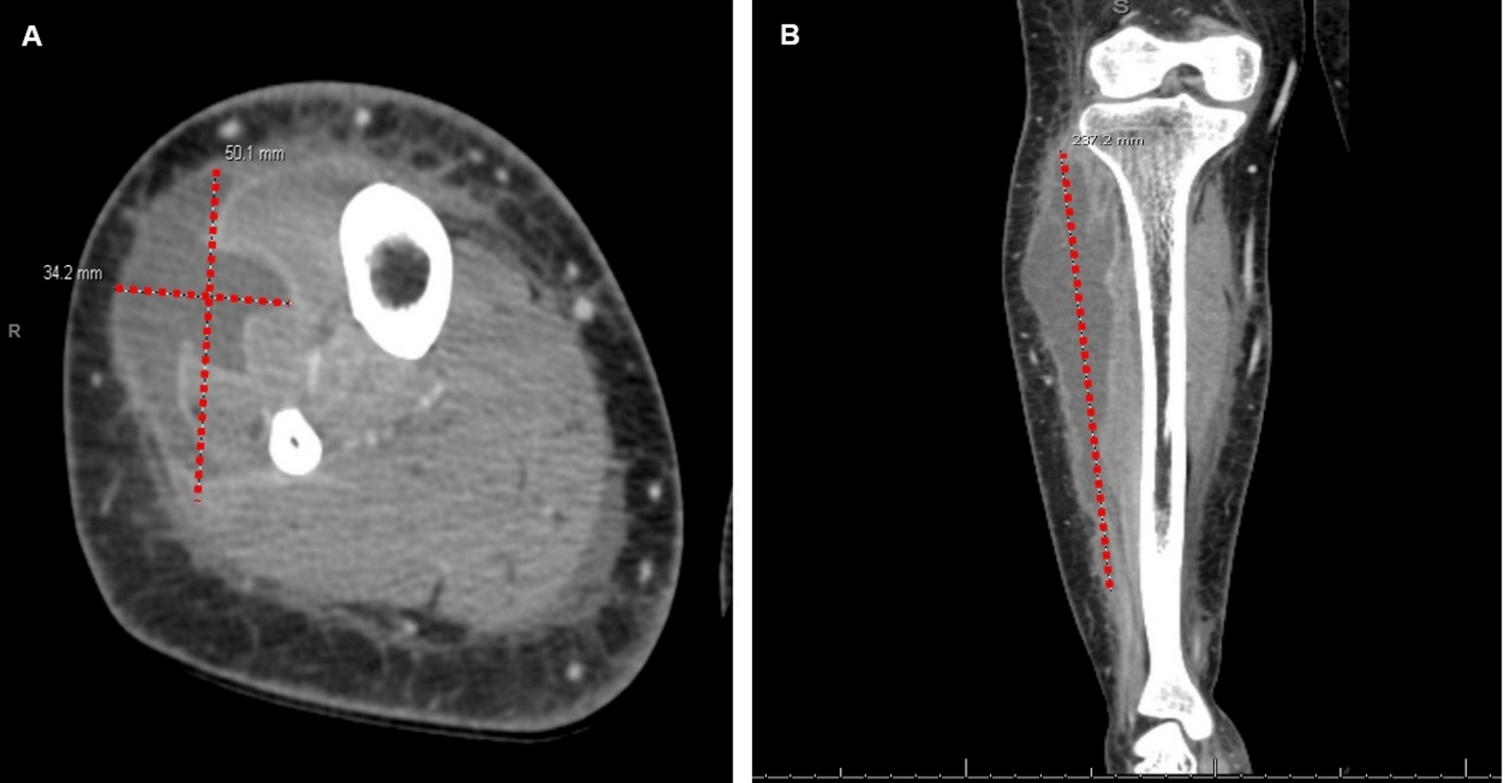
Contrast-enhanced CT of the right lower extremity showing a large multiloculated abscess. Contrast-enhanced CT images of the right lower extremity demonstrate a complex, peripherally enhancing, multiloculated fluid collection within the anterior compartment musculature, measuring approximately 5.0 × 3.4 × 23.7 cm and tapering near the right ankle. Subcutaneous fat stranding is present, with no evidence of gas, cortical erosion, or joint effusion. These findings are consistent with infectious myositis complicated by abscess formation. A includes intersecting red dotted lines marking the central area of the fluid collection in the axial view. B shows the full longitudinal extent of the abscess in the coronal plane. CT: computed tomography

The patient continued to experience significant fatigue, pain, and difficulty bearing weight on the affected leg. Notably, CPK levels remained within normal limits. On hospital day 3, persistent lower extremity pain prompted repeat imaging, this time contrast-enhanced CT imaging of the leg. CT with contrast revealed a 5.0 × 3.4 × 23.7 cm peripherally enhancing, complex, multiloculated fluid collection in the anterior compartment that gradually tapered near the level of the right ankle (Figure 2). Intravenous vancomycin was promptly initiated.

The patient subsequently underwent surgical incision and drainage of the large abscess, with approximately 30 mL of purulent fluid drained and sent for culture. The underlying muscle was viable, and the incision was packed to facilitate drainage and secondary healing. Cultures of samples obtained during surgery were positive for methicillin-resistant *S. aureus.*

After surgery, the patient still had persistent fevers despite receiving appropriate antibiotics, which prompted an additional extensive infectious investigation. Repeat blood cultures remained negative, and additional testing, including a BioFire panel, Fungitell assay, cytomegalovirus, and *Pneumocystis jirovecii*, were all negative. Both transthoracic and transesophageal echocardiograms showed no evidence of vegetation. About eight days after the abscess had been drained, a repeat CT showed significant resolution of the previously seen fluid collection in the anterior leg, with a residual 3.2 cm fluid collection in the distal leg and punctate foci of soft tissue emphysema likely related to postsurgical changes. Re-evaluation by the surgical team found no additional pockets requiring drainage. Therefore, antibiotic therapy was switched to linezolid for two weeks. At the time of discharge, the patient had no fever or pain in the right lower extremity, and the swelling and erythema were considerably improved with successful wound healing and no further complications.

## Discussion

This case highlights the diagnostic challenges of infectious myositis in a young patient on immunosuppressive therapy. The patient presented with localized pain, swelling, and systemic symptoms, but no skin changes. She lacked classic features of a deep muscle infection. Her initial laboratory results were not suggestive of myositis, with a normal CPK and WBC count. The normal CPK levels, despite significant intramuscular infection, may reflect several mechanisms. In immunosuppressed individuals, inflammatory responses and myocyte destruction may be blunted, leading to minimal enzyme leakage. Additionally, in cases where infection is compartmentalized within an abscess rather than diffusely infiltrating muscle fibers, CPK release into circulation may be limited. This paradox highlights a key diagnostic pitfall when relying on traditional biomarkers in this population. Her elevated inflammatory markers could have also been attributed to an SLE flare. These findings delayed recognition of a serious infection. Given the nonspecific presentation and absence of overt signs of infection, several alternative diagnoses were initially considered, including deep vein thrombosis, cellulitis, and an autoimmune flare.

Infectious myositis is rare and classically presents with fever, muscle pain, and swelling, often accompanied by leukocytosis and elevated CPK. However, in immunosuppressed patients, inflammatory markers are unreliable. A retrospective review noted that up to 25% of patients with bacterial myositis may have normal CPK levels and nearly one-third may not exhibit leukocytosis, particularly in those with immunosuppression [2]. Similarly, our patient’s immunosuppressive therapy may have blunted her inflammatory response, masking clinical signs of infection.

Imaging also contributed to the diagnostic delay. The initial non-contrast CT scan suggested soft tissue swelling and possible myositis but did not reveal a discrete abscess. Only when contrast-enhanced CT was obtained later in the hospital course did a multiloculated fluid collection become apparent. The abscess measured 23.7 cm in length, which is substantially larger than what is typically observed in pyomyositis, where abscesses often range from 3 to 8 cm in diameter [4,5]. This unusually large size highlights the extent of disease progression before definitive diagnosis. Contrast-enhanced CT improves detection of fluid collections and abscesses, but magnetic resonance imaging (MRI) remains the gold standard for diagnosing infectious myositis due to its superior soft tissue resolution. MRI can detect early myositis and inflammation even before abscess formation; however, it may not be readily accessible or feasible in acutely ill or unstable patients [6-8]. Additionally, in patients with impaired renal function, the use of gadolinium-based contrast may be contraindicated, further limiting its clinical practicality. Ultrasonography, while useful for superficial infections, is limited in evaluating deep muscle involvement [7,8]. In our case, the patient presented with a creatinine of 3.17 mg/dL, so intravenous contrast was initially avoided. A rapid non-contrast CT in the emergency department provided the necessary soft tissue assessment without adding nephrotoxic risk. After IV hydration improved the patient’s creatinine level, a contrast-enhanced CT was quickly obtained, which then revealed the abscess and guided prompt surgical drainage. MRI was not pursued as contrast-enhanced CT had provided sufficient information for intervention at the time.

Pyomyositis, the formation of abscesses within skeletal muscle due to bacterial infection, was once thought to occur primarily in tropical regions but is now increasingly reported in temperate areas [9]. A population-based study by Maravelas et al. reported a threefold increase in pyomyositis-related hospitalizations in the USA from 2002 to 2014, with *Staphylococcus aureus* being the most frequently isolated pathogen [10]. While skin trauma or local inoculation is often implicated, hematogenous seeding is another well-established mechanism. In our patient, urine cultures were positive for *S. aureus *and *Streptococcus agalactiae*, suggesting that transient bacteremia, possibly undetected by blood cultures, could have led to hematogenous spread and muscle infection. Reports of similar cases support this mechanism. One report described paraspinal and iliacus pyomyositis that developed after a *S. aureus* urinary tract infection, whereas another detailed chest wall pyomyositis caused by *S. agalactiae* [11,12]. Both cases involved immunosuppressed patients with negative blood cultures, and the abscesses were attributed to transient, undetected bacteremia. Given the patient’s immunocompromised status, we suspect a similar mechanism could account for the development of deep muscle abscesses.

The patient’s immunocompromised state due to both SLE and corticosteroid therapy significantly increased her risk for severe and atypical infections. A recent review demonstrated that infections are among the leading causes of hospitalization and mortality in patients with SLE, reflecting vulnerability due to both intrinsic immune dysregulation and treatment-induced immunosuppression [13]. A large national study found that older age, sepsis, and multiple comorbidities are risk factors for poor outcomes in hospitalized patients with SLE and infection; although our patient was young, her case underscores the unpredictable and severe course infections can take in this population [14].

Lastly, surgical management was key in this case. The large abscess ultimately required incision and drainage, although surgical intervention was not initially indicated due to the absence of clear diagnostic findings or systemic instability. At presentation, the source of the patient’s symptoms was unclear, and imaging did not initially reveal a drainable collection. It was only after further clinical progression and repeat imaging that a large, multiloculated abscess was identified, prompting surgical drainage. This underscores the importance of repeat clinical and radiographic assessment when symptoms persist or escalate despite appropriate empiric treatment.

This case illustrates how the absence of classic laboratory or imaging findings should not delay further evaluation in immunocompromised patients with persistent focal symptoms and systemic inflammation. Early recognition, appropriate imaging, and multidisciplinary coordination, including surgical consultation, are critical in preventing morbidity from deep soft tissue infections. Clinicians should maintain a high index of suspicion for infectious myositis in immunosuppressed patients, even when initial workup is inconclusive.

Given the diagnostic uncertainty and potential for rapid progression in this population, structured clinical follow-up or scheduled re-imaging should be considered when symptoms persist despite non-revealing initial studies. This approach may facilitate earlier detection of evolving abscesses or deep infections.

## Conclusions

Early appropriate imaging and clinical vigilance are crucial for accurate diagnosis of infectious myositis in patients who may be immunocompromised for any reason, including use of immunosuppressive therapy for chronic conditions such as SLE. Within this context, laboratory tests may reveal complex results inconsistent with deep tissue infection due to immunosuppression. However, CT with contrast and MRI, when available, are valuable tools for identifying and delineating the extent of infection and guiding treatment decisions. Clinicians should consider any clinical evidence suggesting sources of hematogenous spread in immunocompromised individuals displaying symptoms of muscle infection, such as pain, swelling, and fever, even in the absence of positive blood cultures. This case highlights the importance of integrating evolving inflammatory markers with serial imaging to guide diagnosis and supports earlier consideration of repeat imaging in immunocompromised patients with unexplained, persistent focal symptoms.
